# Exploratory Ecology of Reintroduced Elk in Virginia

**DOI:** 10.3390/ani16121917

**Published:** 2026-06-20

**Authors:** Braiden A. Quinlan, Heather N. Abernathy, David M. Kalb, Jacalyn P. Rosenberger, Emily D. Thorne, William Mark Ford, Michael J. Cherry

**Affiliations:** 1 Department of Fish and Wildlife Conservation, College of Natural Resources and Environment, Virginia Tech, Blacksburg, VA 24060, USA; habernathy@rmef.org (H.N.A.); ethorne@west-inc.com (E.D.T.); 2 Virginia Department of Wildlife Resources, Marion, VA 24354, USA; david.kalb@dem.ri.gov (D.M.K.); jackie.rosenberger@dwr.virginia.gov (J.P.R.); 3 Virginia Cooperative Fish and Wildlife Research Unit, U.S. Geological Survey, Blacksburg, VA 24061, USA; wmford@vt.edu; 4 Caesar Kleberg Wildlife Research Institute, Texas A&M University-Kingsville, 700 University Blvd, MSC 218, Kingsville, TX 78363, USA; michael.cherry@tamuk.edu

**Keywords:** *Cervus canadensis*, home range, movement, reintroduction, site fidelity

## Abstract

Reintroductions of extirpated species are an important tool in wildlife conservation. Understanding how reintroduced populations acclimatize to novel environments can lend insight into social learning that in turn is valuable for assessing reintroduction success and maximizing efficacy of subsequent efforts. During 2012, 2013, and 2014, the Virginia Department of Wildlife Resources implemented soft releases of elk (*Cervus canadensis*) translocated to southwestern Virginia from eastern Kentucky. We investigated home range establishment and post-release movements of these reintroduced elk (*n* = 60). We found adults moved farther from the release site than either yearlings or calves (F = 6.93, *p* = 0.001). Elk released in 2012 and 2013 took similar amounts of time to establish home ranges (median 181 days, range 108–214 days; and median 189 days, range 147–209 days, respectively), but individuals released in 2013 remained closer to the release site ($\bar{x}$ = 605.5 m, SD = 335.7 m, closer) presumably by joining established social groups. However, the 2014 cohort generally took longer to establish home ranges (median: 231 days; range: 56–258 days) and moved farthest from the release site ($\bar{x}$ = 1360.2 m, SD = 293.9 m, farther than 2012 individuals) possibly due to the larger cohort size and resulting intraspecific competition, or the earlier release date that year. Our findings suggest the number of consecutively released cohorts, the timing of the release, and the composition of age classes for released individuals are important considerations for reintroductions.

## Simple Summary

Species reintroductions have become an increasingly common tool for wildlife conservation. Tracking movement tendencies of released animals across the landscape can help inform future reintroductions. During 2012, 2013, and 2014, the Virginia Department of Wildlife Resources released elk (*Cervus canadensis*) captured in Kentucky to southwestern Virginia. We investigated home range establishment and post-release movements of these reintroduced elk. We found adults moved farther from the release site than either yearlings or calves. Elk released in 2012 and 2013 took similar amounts of time to establish home ranges, but individuals released in 2013 remained closer to the release site presumably by joining established social groups. However, elk released in 2014 generally took longer to establish home ranges and moved farthest from the release site possibly due to the larger cohort size and resulting competition, or the earlier release date that year. Our findings suggest the number of released groups, the timing of the release, and the age class structure are important considerations for reintroductions.

## 1. Introduction

Species reintroductions are important tools for wildlife management, conservation biology, and restoration ecology. However, a major challenge in species restoration is insufficient knowledge about habitat requirements and movement ecology relative to the reintroduction area [1]. Understanding how reintroduced animals gather information and establish ranges is key to predicting the rate and scope of population expansion following reintroductions. However, exploratory behavior and drivers of range establishment may be unknown for species being reintroduced into areas that differ from historical conditions in the region or the contemporary conditions from which the animals were sourced. Moreover, when restoration zones are mosaicked by human development, insufficient knowledge of the spatial ecology of reintroduced species can compound social or political conflict associated with reintroduction efforts [2,3], which can undermine restoration success [4,5]. Thus, understanding how animals acclimatize to novel, and by extension, anthropogenically altered, environments is critical, not only for making informed management decisions that increase the probability of success but also for preventing conflicts with stakeholders in restoration zones.

Social learning, or the transmission of information through learned behaviors from conspecifics [6], is the basis for animal culture [7]. Social learning is more associated with herding, or grouping species, and has been observed across avian and mammalian taxa. Observed learned behaviors span tool use, foraging strategies, and vocalizations [7,8]. In ungulates specifically, the act of migration, and the routes taken, have provided the opportunity for analyzing social learning [6,9]. For non-migratory ungulate populations, foraging behaviors are influenced by social learning, but their use as a proxy for studying social learning has often proven inconclusive [10]. Recent work has examined social behaviors of individuals as they exit holding pens [11], but there remains a large gap in research between these small-scale individual behaviors and population-wide movements. With the rise in species reintroductions, post-release movements paired with range establishment on a novel landscape may offer insights into social learning as a new avenue for study.

Although many reintroduction efforts take place across multiple years and often by releasing groups of individuals at discrete timescales, there is little research on how this practice influences reintroduction ‘success’ [12]. ‘Success’ is often context- and site-specific; however, it generally entails maximizing population health and growth while minimizing conflict. Typically, this method is the default due to project funds, public input, and the dynamics/demographics of the source population for the reintroduction [13,14]. Individuals may respond differently to reintroduction if there are conspecifics in the area, particularly if the species is more social or territorial. Cohorts released in years following initial restoration may exhibit different movement strategies while depending on local knowledge of the landscape from prior released conspecifics [15]. For more social species like North American elk (*Cervus canadensis*, hereinafter ‘elk’), individuals typically join social groups, although this is most commonly viewed as an anti-predator strategy [16]. Since elk congregate, having conspecifics already established in the area may increase release site fidelity and decrease time to home range establishment.

Elk had been absent from the central Appalachian Mountains since the mid-1800s as a result of overharvest and habitat loss [17]. At the turn of the 21st century, concerted efforts began to return elk to the region led by Kentucky [17,18] which sourced elk from across the western United States [19]. Following the success of Kentucky’s elk reintroduction, neighboring states including Virginia implemented their own restorations, sourcing individuals from Kentucky due to their proximity [18]. Reintroductions often translocate individuals to unfamiliar areas where the environment may be similar, but the distribution of resources is unknown. Release sites are frequently chosen based on perceptions of high-quality habitat for the released species [14] and minimizing human conflict [2,13,20]. Dispersal from the release site may place animals into lower-quality habitats, which may reduce fitness potential and decrease population growth, ultimately inhibiting reintroduction success [21]. Furthermore, capture, transportation, and release methods affect stress levels and influence initial behaviors and post-release movements as individuals must familiarize themselves with their new landscape for survival [22].

Movements of elk following reintroductions have varied, driven by habitat (release site location), presence of predators, human disturbance, and release methods, as well as being further influenced by age class and sex [12,21,23]. Typically, adults and males move farther from release sites than yearlings, calves, and females [12,21,24]. Research has shown ‘soft releases,’ or those that hold elk in acclimation pens for a period of time following transportation prior to their full release, result in higher release site fidelity than ‘hard releases,’ or cohorts released onto the landscape immediately following transportation [12,21,23]. Further, elk release sites with open areas for foraging and higher densities of edge habitat for cover promote greater release site fidelity [21,23].

Human–elk conflict may arise through vehicle collisions, deleterious herbivory on native plant communities and agricultural crops, and disease transmission to and competition with livestock [2,25,26]. Conflicts are often most pronounced shortly after release due to animals exploring beyond the release site and people getting accustomed to individuals on the landscape [12]. Oftentimes, agricultural producers and landowners adjacent to reintroduction areas are disproportionately affected by elk reintroductions [3,26]. Release methods and knowledge of post-release movement tendencies inform requirements for release site sizes and reduce uncertainty surrounding timing and extent of exploratory movements to adjacent properties [12]. This information is particularly important for the planning stages to ease tension and apprehension of the reintroduction process for stakeholders. Although the Virginia Elk Management Plan [20] provided methods to reduce or eliminate elk conflicts as they might occur, empirical data on post-release behaviors has not yet been quantified for management inclusion.

Information on post-release behaviors, particularly movements, of reintroduced elk would aid in understanding behavior at current and future reintroduction sites. However, this aspect of reintroductions is difficult to quantify because reintroductions are often not experimental, which limits our ability to learn from experience, and elk management is frequently contextual and site-specific [2]. As a result, our objectives were to investigate movement and space use of reintroduced elk and their offspring following release. We hypothesized that home range establishment would take longer for animals released during the first year of the reintroduction given that animals need time to explore and adjust to the unfamiliar landscape (H1). Thus, we predicted that elk released during the first year would take longer to establish their range compared to individuals released in subsequent years (H1:P1). Similarly, we hypothesized that exploratory movements would be the greatest during the first year of the reintroduction given that animals need time to explore the unfamiliar landscape without conspecifics present in the area (H2). Thus, we predicted that elk released during the first year would have greater displacement and exploratory movement patterns compared to individuals released in subsequent years (H2:P1), and exploratory movements (i.e., distance from the release site and weekly displacement) would decrease with each release year (H2:P2). Lastly, we hypothesized that movement patterns would follow seasonal cues and shifts in energetic demands (H3). Thus, we predicted that movements would peak leading up to parturition and during the breeding season (H3:P1).

## 2. Materials and Methods

No artificial intelligence tools were used in this research.

### 2.1. Study Area

**Figure 1:**
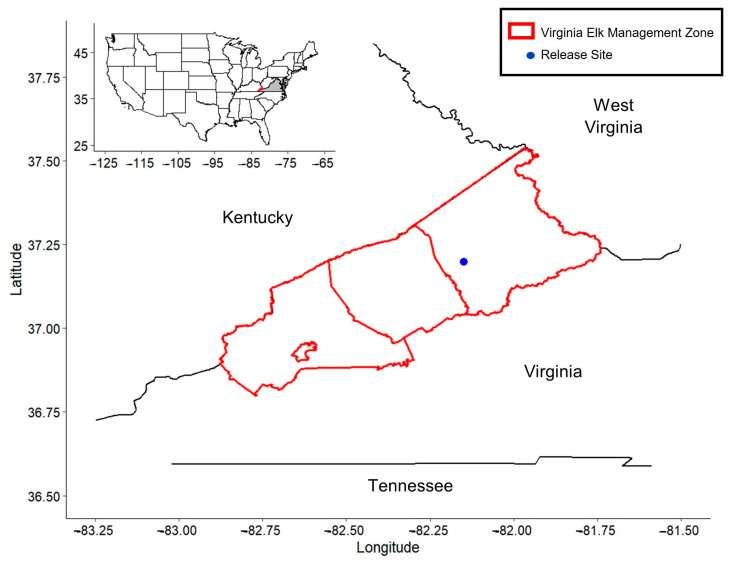
Our study area was the Virginia Elk Management Zone (from left to right in northeast direction: Wise, Dickenson, and Buchanan counties, southwestern Virginia, USA) covering 3220 km^2^ in the central Appalachian Mountains, 2012–2014. The elk release site (blue dot) for each year was in the western portion of Buchanan County.

Our study area was the Virginia Elk Management Zone (VEMZ; latitude: 36.75–37.50, longitude: −83.25–−81.60; Figure 1) covering approximately 3220 km^2^. The VEMZ is situated at the transition of the Appalachian Ridge and Valley to the Appalachian Plateau physiographic sub-provinces within the central Appalachian Mountains Coalfields region (hereafter ‘Coalfields’ [27]). The Coalfields acquired their namesake as a result of widespread subsurface and surface coal mining practices that have greatly altered the natural landscape and many ecological processes therein [28].

This area contained primarily contiguous deciduous forests with limited, interspersed open areas [29,30]. Forests were dominated by American beech (*Fagus grandifolia*), basswood (*Tilia americana*), black cherry (*Prunus serotina*), maples (*Acer* spp.), oaks (*Quercus* spp.), hickories (*Carya* spp.), white ash (*Fraxinus americana*), yellow poplar (*Liriodendron tulipifera*), and some coniferous stands dominated by eastern white pine (*Pinus strobus*; Ref. [29]). Open areas were primarily livestock pastures, hayfields, row croplands, and reclaimed surface coal mines. Livestock pastures were generally a monoculture of non-native tall fescue (*Festuca arundinacea*) whereas reclaimed mines had a larger array of species including non-native grasses and legumes including redtop (*Agrostis alba*), tall fescue, lespedeza (*Lespedeza cuneata*), red clover (*Trifolium pratense*), white clover (*Trifolium repens*), foxtail millet (*Setaria italica*), rye (*Secale cereale*), sweet clovers *(Melilotus* spp.), cat/orchard grass (*Dactylis glomerata*), timothy (*Phleum pratense*), and bird’s-foot trefoil (*Lotus corniculatus*), and several woody plants including eastern white pine, black locust (*Robinia pseudoacacia*), and the non-native autumn olive (*Elaeagnus umbellate* [31,32,33]. The topography in this region is characterized by steep slopes and narrow, incised valleys. Due to surface coal mining practices, some ridgetops have been altered to flat, mesa-like benches and have largely remained that way following reclamation [33].

### 2.2. Collar Data and Monitoring

Groups of elk were captured in Kentucky in 2012 (*n* = 20), in 2013 (*n* = 10) and 2014 (*n* = 45) and held for disease surveillance and health testing. Following a quarantine period of 90 days in 2012 and 2013 which was reduced to a standard 45 days in 2014, elk were transported to a soft release holding pen in Buchannan County, Virginia for approximately two weeks. Release dates were between 23 May and 31 May in 2012, on 5 June in 2013, and 17 April in 2014. The quarantine period was shortened in 2014 because 45 days was deemed sufficient, which resulted in an earlier soft release. Prior to full release, the Virginia Department of Wildlife Resources fit all adult animals and some yearlings and calves with global positioning system (GPS) telemetry collars (ATS G5-2D Iridium, ATS, Isanti, MN, USA) to track movements. Our GPS collars had fix rates of eight hours in 2012 and five hours in 2013 and 2014. Fix rates were in 2013 and 2014 because the conservative fix rate for 2012 was overcautious for battery longevity. Our total elk GPS dataset included adult females (2+ years old; *n* = 29), yearlings (1–2 years old; *n* = 11; F = 1, M = 10), and calves (<1 year old; *n* = 20, F = 9, M = 11). At the time of data collection, there was not a hunting season for elk within the VEMZ.

### 2.3. Ranging Behavior

To test our hypothesis that home range establishment would take longer for animals released during the first year of the reintroduction (H1), we analyzed yearly establishment patterns for animals that had survived for at least six months after their release. We partitioned GPS telemetry data for elk into weekly intervals. Subsequently, we generated weekly auto-correlated kernel density utilization distributions, that we refer to as ‘weekly home ranges’, using the ctmm package, version 1.1.0 [34]. Additionally, we computed ‘accruing seasonal home ranges’ by incorporating location data from all preceding weeks to create a cumulative seasonal range (hereafter accruing seasonal home range). Due to variations in release dates across different years, the start of the weekly accruing home ranges began based on the soft release date from the holding pens across years. In 2012 and 2013, the first weekly home range was between 7 June and 15 June. However, 2014 was calculated using the data collected between 18 April and 27 April given the differences in release dates across years. To quantify home range establishment, we used the Bhattacharyya coefficient which ranges from no overlap (0) to complete overlap (1) using the ctmm package, version 1.1.0 [34]. We considered home range establishment to have occurred when both the weekly home range and the accumulating seasonal home range achieved a Bhattacharyya coefficient value of 1.0. Finally, we constructed linear models with time to home range establishment as the response and year of release as the predictor variable to test our prediction that elk released during the first year would establish their seasonal range later in the year compared to individuals released in subsequent years. These models were developed using the lme4 and lmerTest packages [35,36], versions 1.1-31 and 3.1-3. We used the Akaike information criterion corrected for small sample sizes to determine our top model [37].

### 2.4. Movement Behavior

In null models, movement metrics do not depend on any specific variables included in the model. The movement metric is constant and does not vary based on any measured predictors or covariates. To test our hypotheses that exploratory movements would be the greatest during the first year of the reintroduction and that distance of animals from the release site would progressively decrease as the restoration program advanced (H2), we quantified exploratory movement behaviors post-release. Furthermore, we aligned these movement behaviors with the yearly biological cycle to compare how movements may fluctuate with biological needs. We first partitioned our data into ‘weeks since release’ wherein the start date began based on release dates specific to each individual. Next, we calculated the net-squared displacement (NSD) for several movement metrics. NSD is a widely employed method for examining individual movement strategies over time, as it captures fundamental aspects of animal movement by measuring the square of the Euclidean distance between an individual’s initial location and each successive location along their movement trajectory [38,39]. Specifically, we calculated NSD values in three distinct ways: (1) weekly displacement from the soft release site (SRS), (2) weekly displacement between the starting and ending locations each week, and (3) weekly displacement maximums. We also calculated average weekly movement rates between all locations at the starting and ending location of the week. Weekly speed was estimated by dividing distance between sequential points by time between sequential points wherein units are in meters per hour.

We anticipated non-linear effects on movements associated with reproductive chronology; therefore, we implemented generalized additive models (GAMs) using the mgcv package (version 1.8-23 [40,41]). By incorporating smoothing functions, GAMs offer greater flexibility in capturing non-linear relationships between predictors and the response variable [42,43]. To balance model fit to the data with model complexity to prevent overfitting, we used generalized cross validation (GCV) to select our smoothing parameter [40,44]. To test our prediction that elk released during the first year would have greater weekly displacement and exploratory movement patterns compared to individuals releases in subsequent years (H2:P1), we created three competing sets of GAMs for the dependent variables NSD average, NSD maximums, and speed. In each competing set, we constructed models predicting NSD metrics and speed of all possible combinations, with increasing weeks since release and Julian date as smoothing terms and age class (calf, yearling, adult), and release year (2012, 2013, and 2014) as the grouping or conditioning variable on the smoothing terms. To directly test our H2, the year of release served as our linear predictor variable in all models, except the intercept-only one, across competing sets.

To further test our prediction that distance between animals and the SRS would progressively decrease as the restoration program advanced from 2012 to 2014 (H2:P2), we constructed two competing sets of GAMs for the dependent variables average weekly NSD from the SRS and average weekly distance from the SRS. In this case, we generated models of all possible combinations, with increasing weeks since release and Julian date as smoothing terms and age class (calf, yearling, adult), and release year (2012, 2013, and 2014) as the grouping or conditioning variable on the smoothing terms. To directly test our second hypothesis, the year of release served as our linear predictor variable in all models, except the intercept-only one, across competing sets. In all our GAMs, we included model weights which correspond to the number of GPS locations collected by each animal during each ‘week since release’. For each candidate set we used the Akaike information criterion corrected for small sample sizes to determine our top model (AICc; Ref. [37]).

To explore our third hypothesis and its prediction that movement patterns would follow seasonal cues and shifts in energetic demands such as peaking leading up to parturition and during the breeding season (H3:P1), we compared NSD across Julian days. Specifically, we considered peak parturition activity to take place in late May to early June (around Julian day 152) and peak breeding activity to take place in late September into October (around Julian day 275). All data manipulation and analyses were conducted in Program R version 4.3.1 [45].

## 3. Results

### 3.1. Ranging Behavior

We analyzed data from 60 elk (2012, *n* = 9 females; 2013, *n* = 10 [2 females, 8 males]; 2014, *n* = 41 [28 females, 13 males]) that had 55,070 recorded GPS locations from 2012 to 2014 (2012, *n* = 4190; 2013, *n* = 9236; 2014, *n* = 41,644). We removed 16 animals from our data that did not have sufficient sampling to estimate home ranges. Average lifetime home range size among remaining animals (*n* = 44 [2012, *n* = 6; 2013, *n* = 10; 2014, *n* = 28]) across all years was 10.06 km^2^ (range: 7.65 to 12.88 km^2^).

**Figure 2:**
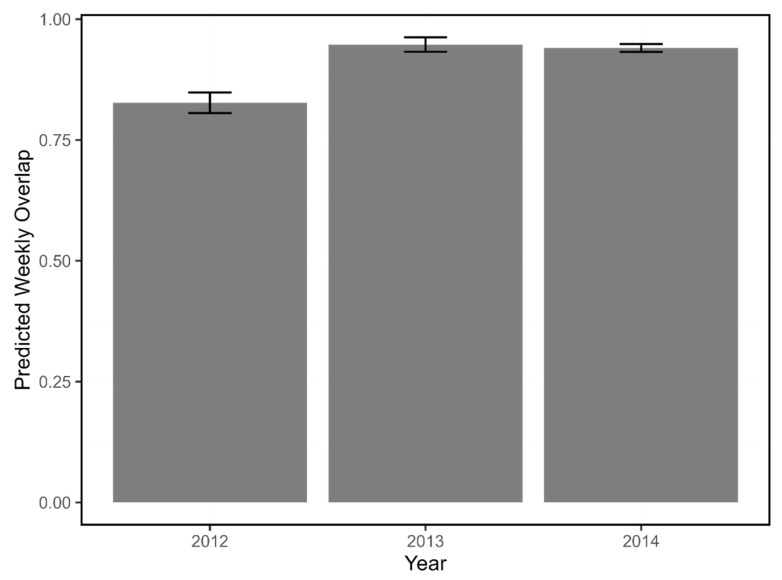
Predictions from our top model of weekly accruing home range overlap of reintroduced elk (*Cervus canadensis*) in southwestern Virginia from 2012 (*n* = 9), 2013 (*n* = 10) and 2014 (*n* = 41). Bars above and below each bar represent the upper and lower 95% confidence intervals.

Year of release appeared to influence home range establishment as our hypothesis-driven model (H1) performed better than the null model (Table A1). Animals released in 2012 had less home range overlap, on average, each week (83%, CI: 81–85%) compared to animals released in 2013 (95%, CI: 92–98%) and 2014 (94%, CI: 92–97%) (Table A2; Figure 2). Notably, the year of release not only influenced the degree of weekly home range overlap but also manifested in distinct temporal dynamics related to home range establishment. Animals released in year one, between 23 May and 31 May 2012, attained an overlap value of 1.0 for accruing home ranges at 181 days after release (range: 108–214 days). Individuals released in year two, on 5 June 2013, reached an overlap value of 1.0 for accruing home ranges at 189 days after release (range: 147–209 days). Animals released in year three, on 17 April 2014, reached an overlap value of 1.0 for accruing home ranges at 231 days after release (range: 56–258 days). These temporal patterns suggest that the year of release not only influences the extent of overlap but also impacts the timing at which animals establish their home ranges (Table A2).

### 3.2. Movement Behavior

**Figure 3:**
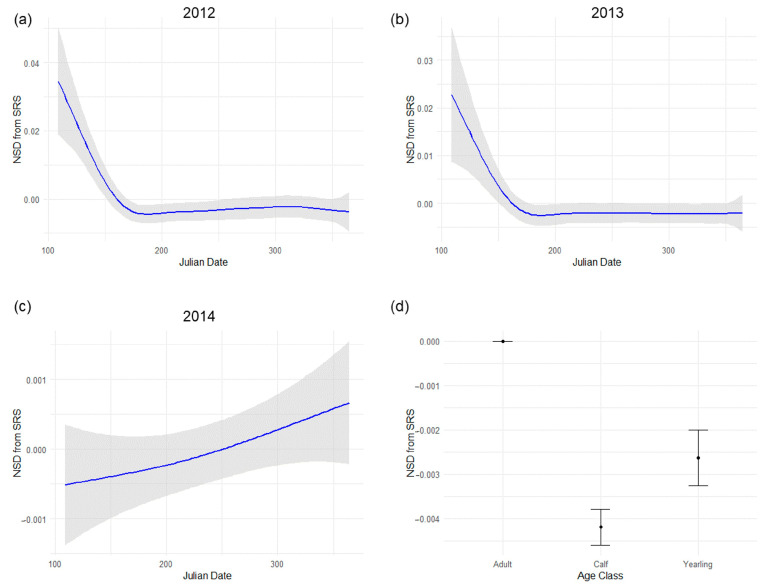
Net-squared displacement (NSD) from the soft release site (SRS), with 0 as the reference, of elk (*Cervus canadensis*) in southwestern Virginia for each release year, 2012 [(**a**); *n* = 9], 2013 [(**b**); *n* = 10], and 2014 [(**c**); *n* = 41], based on Julian date. Blue lines are the calculated NSD, and gray shaded areas are the 95% confidence intervals. The bottom right plot (**d**) depicts the partial residuals for the three age classes (adult, calf, and yearling) with 95% confidence intervals.

**Figure 4:**
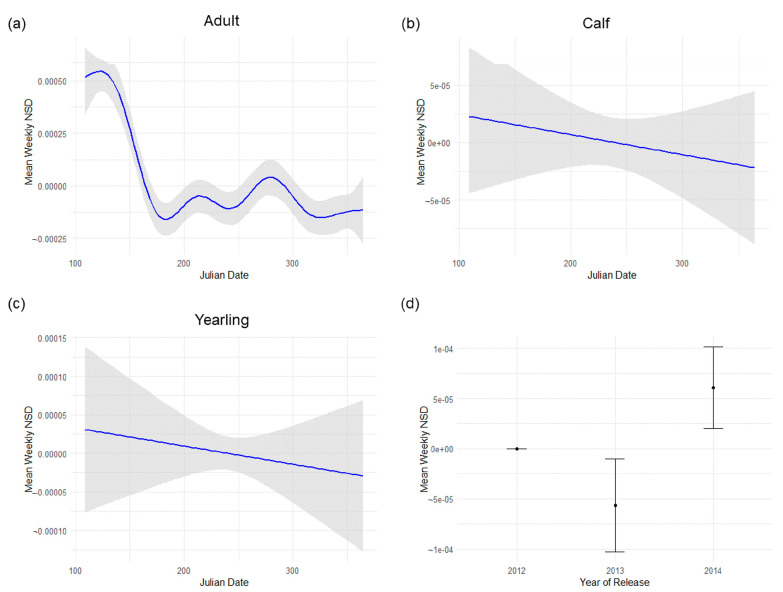
Mean weekly net-squared displacement (NSD), with 0 as the reference, for elk (*Cervus canadensis*) in southwestern Virginia from 2012 (*n* = 9), 2013 (*n* = 10), and 2014 (*n* = 41) based on age class: adult (**a**), yearling (**b**), and calf (**c**). Blue lines are the calculated weekly mean NSD, and gray shaded areas are the 95% confidence intervals. Parturition and breeding seasons occur around Julian dates 160 and 275, respectively. The bottom right plot (**d**) depicts the partial residuals for the three release years (2012, 2013, and 2014) with 95% confidence intervals.

**Figure 5:**
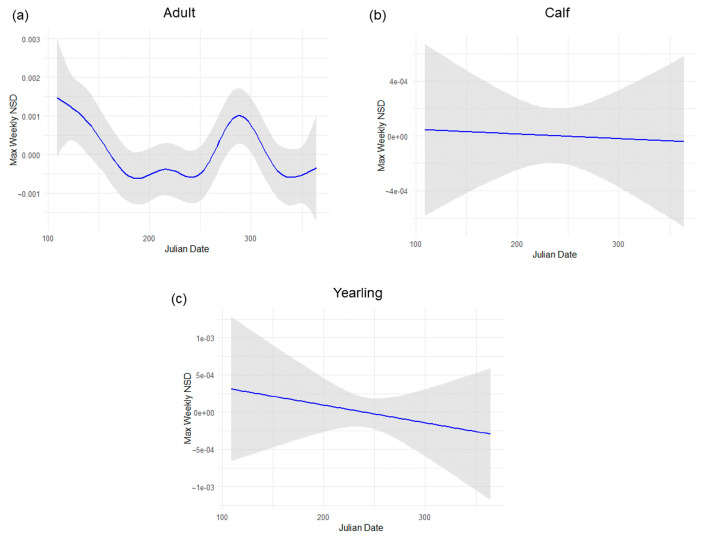
Weekly maximum net-squared displacement (NSD), with 0 as the reference, for elk (*Cervus canadensis*) in southwestern Virginia from 2012 (*n* = 9), 2013 (*n* = 10), and 2014 (*n* = 41) based on age class: adult (**a**), yearling (**b**), and calf (**c**). Blue lines are the calculated weekly maximum NSD, and gray shaded areas are the 95% confidence intervals. Weekly maximum NSD was measured in kilometers using that week’s origin location. Parturition and breeding seasons occur around Julian dates 160 and 275, respectively.

Movement behavior happened at a finer scale than ranging behavior, so an animal that lost its collar or died prior to establishing a home range could still provide data for movement behavior. Therefore, we included data from all 60 collared elk when analyzing movement behaviors. The greatest variation in NSD was between Julian days 100 and 365. We also observed a spike in mean and maximum NSD occurring between Julian days 250 and 330, which loosely correlates to the date range of early September (6 September in 2012 and 7 September in 2013 and 2014) to 26 November (Figure 3, Figure 4 and Figure 5). Our H2 models performed better than the null model (Table A3), and weekly displacement rates in the first year of release (2012) were significantly different than those for individuals released in 2013 and 2014 (Table A4). Further, movements of elk fluctuated throughout the year, reflected by the significance of the smoothing term ‘Julian date’ and its relatively high complexity (effective degrees of freedom; EDF: 8.08; Table A4), and suggested a relatively complex non-parametric relationship between Julian date and weekly NSD rates for adults (*p* < 0.001; Table A4). In contrast, calves and yearlings showed less fluctuations in movements throughout the year, suggesting the relationship driving NSD is less dynamic for these age groups (Table A4). The model explained 9.39% of the variation in mean net displacement (Adjusted R-squared: 0.093), suggesting that other unaccounted factors (e.g., reproductive chronology) contributed to the observed variability in mean NSD.

Our top model for our second hypothesis (H2), that exploratory movements would be the greatest during the first year of the reintroduction, performed better than the null model (Table A5). We found maximum weekly displacement rates in the first year of release (2012) were not significantly different than those for individuals released in 2013 and 2014 (Table A6). However, maximum weekly displacement of adults fluctuated throughout the year (*p* = 0.009) as shown through the significance of the smoothing term Julian date and its relatively high complexity (EDF: 6.74; Table A6). Again, maximum weekly displacement did not fluctuate as much for calves and yearlings (Table A6). That model explained very little of the variation in maximum net displacement (Adjusted R-squared: 0.0047), suggesting factors besides age class contributed more to maximum weekly displacement events.

**Figure 6:**
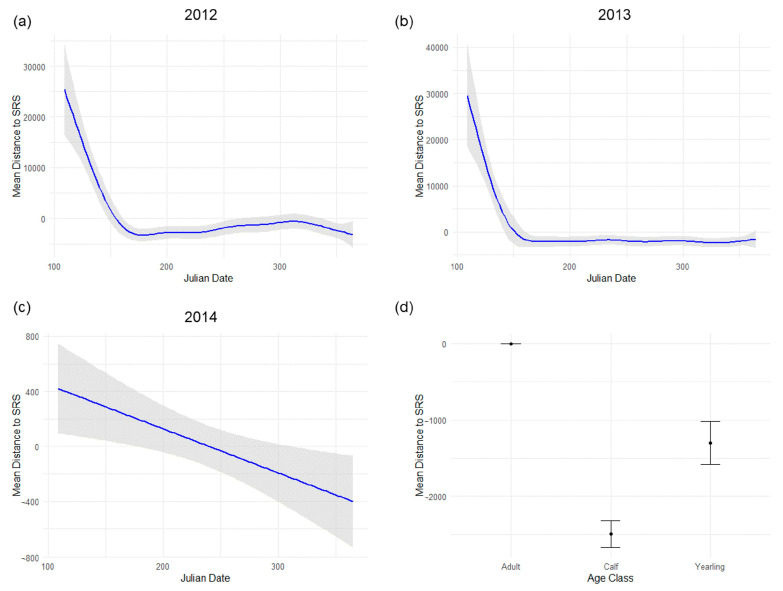
Mean weekly speed, with 0 as the reference, for elk (*Cervus canadensis*) in southwestern Virginia for each release year, 2012 [(**a**); *n* = 9], 2013 [(**b**); *n* = 10], and 2014 [(**c**); *n* = 41], based on Julian date. Blue lines are the estimated mean speed, and gray shaded areas are the 95% confidence intervals, both measured in meters per hour. Parturition and breeding seasons occur around Julian dates 160 and 275, respectively. The bottom right plot (**d**) depicts the partial residuals for the three age classes (adult, calf, and yearling) with 95% confidence intervals in which the adult age class is the baseline for comparisons with the other two age classes in meters per hour.

For weekly movement rates, our H2 model performed better than the null model (Table A7) and suggested that weekly speeds (average meters per hour) in the first year of release (2012) were significantly different than those for individuals released in 2013 and 2014 (Figure 6; Table A8). Relative to 2012, weekly speeds of animals released in 2013 and 2014 were on average 65 and 57 m per hour slower (Table A8). Moreover, adults had the greatest fluctuation in weekly movement rates throughout the year, followed by calves and then yearlings (EDFs for adults: 8.28, calves: 6.78, yearlings: 3.39; Table A8). However, other factors outside the year of release and age class, such as reproductive chronology, likely contribute to weekly movement rates as well (Adjusted R-squared: 0.168).

**Figure 7:**
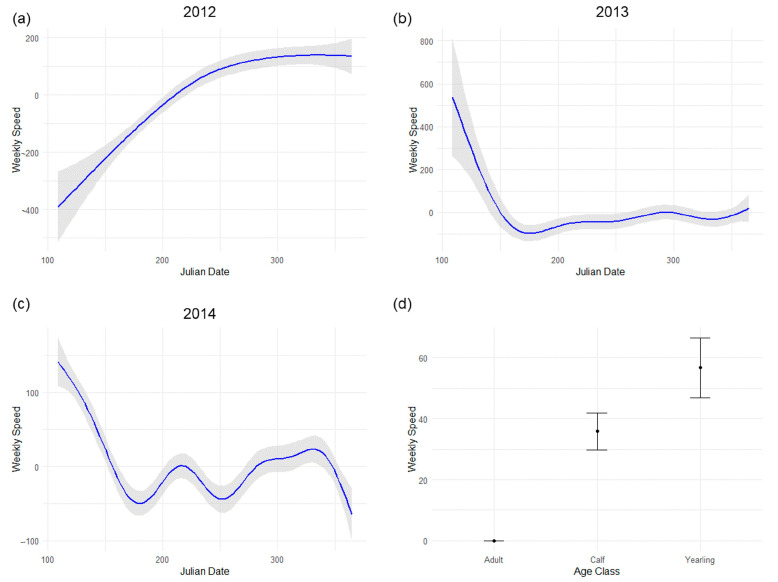
Mean distance, with 0 as the reference, to the soft release site (SRS, meters) for elk (*Cervus canadensis*) in southwestern Virginia for each release year, 2012 [(**a**); *n* = 9], 2013 [(**b**); *n* = 10], and 2014 [(**c**); *n* = 41], based on Julian date. Blue lines are the calculated mean distance to the soft release site, and gray shaded areas are the 95% confidence intervals measured in meters. Parturition and breeding seasons occur around Julian dates 160 and 275, respectively. The bottom right plot (**d**) depicts the partial residuals for the three age classes (adult, calf, and yearling) with 95% confidence intervals.

Our top model for our second hypothesis (H2:P2), that displacement from the SRS would decrease, performed better than the null model (Table A9) and suggested the distance between animals and the SRS increased as the restoration program advanced from 2012 to 2014 (Figure 7; Table A10). Relative to 2012, animals released in 2014 tended to have greater weekly displacement rates from the SRS (Table A10). Moreover, the adult age class again showed the greatest fluctuations in weekly displacement rates from the SRS throughout the year (EDF for adults: 1.66, Table A5). Similarly, our top model for H2 (Table A11) showed that relative to 2012, animals released in 2014 were farther from the SRS, and while marginally significant, those in 2013 were somewhat closer to the SRS compared to 2012 (Table A12). Surprisingly, calf distance from SRS fluctuated throughout the year (F = 7.91, Table A12).

Lastly, comparing movements throughout the year (Julian days) revealed fluctuations reflected by reproductive chronology (H3:P1). We observed increases in movement metrics (NSD and speed) during the onset of parturition and during the breeding season. Paired with these increases in movements were decreases between and after them.

## 4. Discussion

We found animals released in the final year of restoration displaced farther within the first 30 weeks post-release, compared to those released in years one and two. Consequently, individuals released in the last release year, 2014, took the longest to establish home ranges. Moreover, we found differences among age classes in movement patterns with adults dispersing farthest from the SRS followed by yearlings with calves remaining closest. We found an inflated movement period for individuals immediately following their release that decreased in subsequent years. Our analyses suggested elk exhibited variation in weekly movement and space use by release year that also coincided with the onset of biological seasons such as the mating season (rut) and parturition.

Unexpectedly, founding animals did not take longer to establish home ranges compared to animals released in the subsequent release years. Across many taxa, home range establishment and overlap are used as a proxy for social learning and behavior, largely through the lens of territories [46]. In territorial social species, members of social groups learn territories from conspecifics and dominant individuals [46,47]. These territories provide social groups with adequate forage resources, thereby minimizing interactions with competing social groups [48]. Further, social groups can promote the faster diffusion of information such as foraging areas and strategies [49]. While elk are not territorial, they are social year-round. We observed greater home range overlap (Figure 2) amongst individuals in subsequent release years consistent with other cervid species such as the Persian fallow deer (*Dama mesopotamica* [50,51]). Greater range overlap and a shorter initial dispersal period could be the result of elk joining established social groups from previous release years and utilizing their knowledge of the novel landscape to exploit higher quality habitats.

Contrary to our predictions, the 2014 cohort traveled the farthest from the SRS and took the longest to establish home ranges (Figure 3 and Figure 7). Our results may have been confounded by differing times of release among years. The 2012 and 2013 releases took place at the onset of the parturition period for elk (late May and early June, respectively), whereas the 2014 release occurred approximately four weeks earlier in the year (mid-April). Releasing pregnant females close to the parturition window leverages philopatry and is believed to anchor elk closer to the release site which is likely a strong modifier to their post-release movements as observed with other elk release efforts [12,21]. We posit that elk released in 2014 had more time to explore and become familiar with their new environment without the immediacy of parturition that might otherwise curtail movement [12,52,53]. Further, 2014 was the largest cohort released in Virginia (approximately twice the size of 2012 and four times the size of 2013). Accordingly, it is possible that this larger cohort perceived more potential intraspecific competition upon release and displaced themselves farther from the SRS as a result. We did not include weather (i.e., temperature and precipitation) in our models as a driver of movements from year to year. Both 2012 and 2013 had similar monthly average temperatures and precipitation during the months of the release and immediately following them [54]. However, 2014 had consistently higher average temperatures (over 2 °F warmer monthly averages than 2012 and 2013) and less precipitation (approximately four inches less than the 2012 and 2013 average during the three months following release) [54]. These warmer and drier conditions may also have increased the effect of intraspecific competition for the 2014 cohort due to the poorer plant growing conditions.

Elk released in Virginia were subjected to ‘moderately soft’ releases based on their holding period of approximately two weeks for each release year. Other studies of elk reintroductions have shown that longer periods of in situ acclimation leads to higher site fidelity, or a tendency for released animals to remain near the release site. Bleisch et al. [12] found that six months after release, 83% of elk held for 129–163 days before reintroduction to the Missouri Ozarks remained within 10 km of their release site. Similarly, Ryckman et al. [23] found elk reintroduced into Ontario, Canada, that were held for short periods (4–11 days) dispersed relatively far from the release site (22.6–26 km), whereas most elk (70%) held for longer periods (17–112 days) remained near the release site (7.3–17.5 km). Post-release dispersal was also strongly negatively related to the length of the ‘soft release’ period in reintroduced Key deer (*Odocoileus virginianus clavium* [55]). Furthermore, if individuals continue to disperse farther from their release site immediately following their reintroduction, it may decrease the density of the population to a level that hinders herd cohesion and sociality and therefore potential population growth and stability [21,56]. Our results paired with these other studies demonstrate the importance of holding periods for release efforts in relation to release site fidelity, particularly for the founding cohort if multiple release efforts are implemented.

Movements by cervids are often associated with seasonal variation in an individual’s metabolic requirements and forage availability and quality [24,57,58]. We observed periods of greater movements coinciding with the onset of different biological seasons, specifically leading to parturition and the breeding season. Our study area experiences a decrease in forage availability during the winter dormant season [59]. However, milder winters in our study area (higher temperatures and less snowfall) result in year-round local forage availability that does not necessitate migratory movements to optimize forage availability when compared to migratory western elk populations habituating more drastic seasonality (e.g., the Rocky Mountains) [58]. Still, on a smaller, local scale, elk may move to utilize specific habitat patches within their ranges during different times of year. For instance, our study area was dominated by closed-canopy deciduous hardwood forests [29,31,33] that provide limited forage and thermal cover for elk during the dormant season but may be of higher quality during the growing season and summer due to more digestible and abundant forage, respectfully [59,60,61]. Reclaimed surface coal mines are a much smaller proportion of our study area and are distributed as large but highly disjunct patches on the landscape [29,30]. These reclaimed mines generally provide elk with quality habitat throughout the year, supplying year-round forage, thermal refugia, and cover from predators [59,62,63]. This patchy resource paired with milder winters may promote increased movements by elk during this time of year albeit at a smaller scale compared to western counterparts [58].

Parturient females of many ungulate species seclude themselves from conspecifics for parturition [57,64,65]. Isolation serves as a way for mothers to bond with their offspring and take advantage of forage and cover resources better suited for their individual needs [57,66,67,68,69]. In our study, adult female elk had greater movements prior to parturition (2014 cohort) which decreased during and immediately following parturition (late May through June). During this period, mothers are constrained by the limited movements of their offspring, thus decreasing their movements until the calf becomes more mobile. During parturition and calf-rearing, female elk may make tradeoffs between access to quality forage and protective cover [66]. Berg et al. [66] found female elk in Banff National Park, Canada, prioritized high-quality forage, thereby prioritizing their own fitness in the face of higher predation risk from grizzly bears (*Ursus arctos horribilis*), gray wolves (*Canis lupus*), and mountain lions (*Puma concolor*), and thus increasing predation risk for their offspring. In our study area, black bears (*Ursus americanus*) are the only large predators, and they occur at lower densities regionally [56], prompting low associated predation risk for adult females. As a result, postpartum females can instead prioritize protection of their offspring in their early life stages [70,71,72] which may explain the decreased movements during this time of year.

Following the parturition season, net-squared displacement from the SRS for adults increased in early August (Figure 4 and Figure 5). By this time, calves are fully mobile, no longer limiting the movements of their mothers [57,66], allowing mothers to exploit higher quality foraging habitats to meet the high energetic demands of lactation [73,74]. Further, once calves are fully mobile by late summer, new family pairs rejoin matriarchal groups [57,66,70]. Matriarchal groups offer shared care with greater protection of offspring by allowing for more efficient foraging as individuals can take turns being vigilant [71]. These findings paired with our results suggest late summer movements are likely influenced by energetic requirements and life history strategies.

We observed weekly movement rates (both mean and maximum and exploratory behavior around a centralized location) increase from early September to mid/late-October then decrease through November (Figure 4 and Figure 5). In our study area, the breeding season (rut) for elk occurs during early to mid-autumn, peaking in late September to early October. During the rut there is increased activity and movements, particularly for mature males [58,75]. However, our observations revealed increased weekly displacement for adult females whereas yearlings and calves largely reduced their weekly displacement during this time. The energetic demands of the rut are generally explored with males [75,76], but our results suggest adult females may also have elevated energetic demands during this time of year as a result of, or indicated by, their movements. Although results vary amongst ungulate species [24,76,77], one explanation for more pronounced movements by females during the rut could be the high male sex ratio observed in our population [78]. The high ratio of males and the fact that they were all inexperienced breeders (sub-adults) could have increased mating pressure and harassment amongst female conspecifics leading to increased chasing and movements by females. Alternatively, because no mature male elk were released during the study period, females may have increased movements to search for them. We were unable to compare male and female movements due to the distribution of sexes between age classes. It is common for elk reintroductions to release yearling and calf males because sources do not want to remove mature bulls from their populations. Differences between male and female movements and male age classes usually do not reveal themselves until males are over two years old. Prior to two years old, males generally move with the matriarchal groups throughout the year [12,75,79]. In our study, all males released were immature (<2.5 years old). As a result, we did not expect to see differences between male and female movements post-release.

After elk became acclimatized to their environment following their reintroduction, they decreased their movements and generally remained close (within 4 km on average) to the SRS. Adult exploratory behavior across this landscape was significantly greater than other age classes as indicated by increased displacement. It is likely that animals stopped dispersing when they found suitable environments similar to the reclaimed mine habitat in Kentucky where the elk were sourced, or after stress following transportation and release had subsided [22]. Many introduced species demonstrate strong release site fidelity including elk in Wisconsin [24], Missouri [12], Kentucky [21], and North Carolina [80]. Home range sizes for female elk decreased with time since release for each cohort, suggesting familiarity allowed for more efficient space use as observed in mule deer (*Odocoileus hemionus* [81]). Optimal foraging theory and movement economy theory postulate animals exert the least amount of energy possible to meet their metabolic needs, particularly in instances of resource scarcity [82]. Presumably, high-quality habitat around the SRS reduced the need for greater dispersal and continued exploratory movements by reintroduced individuals. In our study, elk were released on a reclaimed surface coal mine [20] which is known to be the predominantly selected elk habitat in the central Appalachian Mountain region [62,63,83,84]. Relative to this Virginia herd, Quinlan et al. [59] observed that the reclaimed mines in Virginia were the highest quality habitat type for elk based on their movements and home ranges during the first decade following reintroduction.

## 5. Conclusions

Our results contribute to the growing understanding of the drivers for exploratory ecology of reintroduced species. Release site selection for reintroductions is important as this area will likely have legacy effects as reintroduced animals will likely return to or remain in the area [21,81]. Our results paired with others [59] found exploratory movements likely occurred as a function of biological season and potentially due to the patchiness of the landscape [59], and thus it is important to consider release sites in relation to surrounding forage and cover. As we and others observed [12,23,58], there was an initial dispersal period for individuals immediately following their release that decreased in the second year of reintroduction. Successively released cohorts may use social cues from established individuals for prioritizing home ranges [50,51].

Further, our results indicate adult elk appear to instigate this initial dispersal across the landscape with yearlings and calves remaining closer to the release site [21,23,24]. Our work supports previous assertions that releasing a higher proportion of calves and yearlings may promote stronger release site fidelity [21,23]. However, this practice may inhibit population growth due to inexperienced breeders and lower proportions of sexually mature individuals. Beyond the demographics of the cohort, we found the timing of the release in relation to the species life history strategies likely plays a large role in their movement ecology. Releasing pregnant females close to their parturition period may effectively ‘anchor’ them to the release site as proposed by others [12,21,24]. These practices could be used to establish herds in specific restoration areas to avoid conflict. If the goal is to populate a more expansive restoration zone quickly, hard releases of many individuals would likely be a better method. Careful considerations are required regardless of the release method chosen based on restoration goals.

## Data Availability

At the time of publication, these data were not publicly available from the Virginia Department of Wildlife Resources. Author Quinlan (braidenq@vt.edu) can be contacted for data availability upon reasonable request.

## Abbreviations

The following abbreviations are used in this manuscript:

AICc	Akaike Information Criterion Corrected for Small Sample Sizes
CI	Confidence Interval
EDF	Effective Degree of Freedom
GAM	Generalized Additive Model
GCV	Generalized Cross Validation
GPS	Global Positioning System
NSD	Net-Squared Displacement
SRS	Soft Release Site
VEMZ	Virginia Elk Management Zone

## Appendix A

**Table A1:** Output from model selection results examining weekly home range overlap of reintroduced elk (*Cervus canadensis*) in southwestern Virginia from 2012 to 2014. We used the Akaike information criterion with a correction for small sample sizes (AICc) to determine if our hypothesis-driven model performed better than the intercept-only model. The table displays the following information for each model: model formulations, number of model parameters (K), AICc values, delta AICc, and the log likelihood (LL).

Model Formulation	K	AICc	Delta AICc	LL
Year of Release	5	−1729.00	0.00	868.51
Intercept-only	3	−1635.02	93.97	819.52

**Table A2:** Output from top linear model examining weekly home range overlap of reintroduced elk (*Cervus canadensis*) in southwestern Virginia from 2012 to 2014. The table displays each model variable and the corresponding variable, beta estimate, lower and upper confidence intervals (CIs), standard error (Std. Error), and t-value. The release year of 2012 is the reference category (Intercept).

Variable	Estimate	Lower 95% CI	Upper 95% CI	Std. Error	t-Value
Intercept	0.83	0.81	0.85	0.01	76.15
year2013	0.12	0.09	0.15	0.01	9.06
year2014	0.11	0.09	0.14	0.01	9.74

**Table A3:** Output from model selection examining weekly mean net-squared displacement (NSD) starting on the date of release for reintroduced elk (*Cervus canadensis*) in southwestern Virginia from 2012 to 2014. We used the Akaike information criterion with a correction for small sample sizes (AICc) to determine which combination of variables best influenced elk movement. The table displays the following information for each model: model formulations, degrees of freedom (DF), AICc values, delta AICc, and the log likelihood (LL). WSR is ‘weeks since release’.

Model Formulation	DF	AICc	Delta.AICc	LL
mean NSD ~ s(julian_date, by = age) + year	14.08	−24,285.34	0.00	12,156.86
mean NSD ~ s(WSR, by = age) + year	13.37	−24,229.99	55.35	12,128.47
mean NSD ~ s(WSR, by = year) + year	12.91	−24,220.29	65.05	12,123.15
mean NSD ~ s(julian_date, by = year) + year	13.32	−24,219.48	65.86	12,123.16
mean NSD ~ 1	2.00	−24,105.01	180.33	12,054.51

**Table A4:** Top model output with approximate significance of smoothing terms for mean net-squared displacement for elk (*Cervus canadensis*) in southwestern Virginia from 2012 to 2014. EDF is effective degrees of freedom and Ref.df is reference degrees of freedom.

**Parametric Coefficients**				
	Estimate	Std. Error	t value	Pr (>|t|)
(Intercept)	1.543 × 10^−4^	3.846 × 10^−5^	4.011	<0.001
year2013	−5.640 × 10^−5^	4.634 × 10^−5^	−1.217	0.224
year2014	6.095 × 10^−5^	4.051 × 10^−5^	1.504	0.133
**Approximate significance of smooth terms**				
	EDF	Ref.df	F	*p*-value
s(julian_date):ageAdult	8.084	8.775	19.787	<0.001
s(julian_date):ageCalf	1.000	1.000	0.497	0.481
s(julian_date):ageYearling	1.000	1.000	0.358	0.550

**Table A5:** Output from model selection examining weekly maximum net-squared displacement (NSD) starting on the date of release for reintroduced elk (*Cervus canadensis*) in southwestern Virginia from 2012 to 2014. We used the Akaike information criterion with a correction for small sample sizes (AICc) to determine which combination of variables best influenced elk movement. The table displays the following information for each model: model formulations, degrees of freedom (DF), AICc values, delta AICc, and the log likelihood (LL). WSR is ‘weeks since release’.

Model Formulation	DF	AICc	Delta.AICc	LL
max NSD ~ s(julian_date, by = age) + year	12.74	−15,546.83	0.00	7786.25
max NSD~ s(WSR, by = age) + year	12.24	−15,542.60	4.23	7783.63
max NSD ~ 1	2.00	−15,540.09	6.75	7772.05
max NSD ~ s(julian_date, by = year) + year	10.27	−15,539.76	7.08	7780.21
max NSD ~ s(WSR, by = year) + year	11.04	−15,539.58	7.25	7780.90

**Table A6:** Top model output with approximate significance of smoothing terms for max weekly net-squared displacement for elk (*Cervus canadensis*) in southwestern Virginia from 2012 to 2014. EDF is effective degrees of freedom and Ref.df is reference degrees of freedom. Std. Error is standard error.

**Parametric Coefficients**				
	Estimate	Std. Error	t value	Pr (>|t|)
(Intercept)	4.736 × 10^−4^	3.597 × 10^−4^	1.316	0.188
year2013	−1.957 × 10^−4^	4.336 × 10^−4^	−0.451	0.652
year2014	2.528 × 10^−4^	3.789 × 10^−4^	0.667	0.505
**Approximate significance of smooth terms**				
	EDF	Ref.df	F	*p*-value
s(julian_date):ageAdult	6.74	7.86	2.64	0.009
s(julian_date):ageCalf	1.00	1.00	0.01	0.936
s(julian_date):ageYearling	1.00	1.00	0.04	0.849

**Table A7:** Output from model selection examining weekly mean speed (m/hour) starting on the date of release for reintroduced elk (*Cervus canadensis*) in southwestern Virginia from 2012 to 2014. We used the Akaike information criterion with a correction for small sample sizes (AICc) to determine which combination of variables best influenced elk movement. The table displays the following information for each model: model formulations, degrees of freedom (DF), AICc values, delta AICc, and the log likelihood (LL). WSR is ‘weeks since release’.

Model Formulation	DF	AICc	Delta.AICc	LL
mean_sl ~ s(julian_date, by = age) + year	22.43	24,341.31	0.00	−12,147.95
mean_sl ~ s(julian_date, by = year) + year	16.62	24,350.45	9.13	−12,158.45
mean_sl ~ s(WSR, by = year) + year	16.50	24,357.26	15.95	−12,161.98
mean_sl ~ s(WSR, by = age) + year	21.09	24,495.67	154.35	−12,226.50
mean_sl ~ 1	2.00	24,680.86	339.55	−12,338.43

**Table A8:** Top model output with approximate significance of smoothing terms for mean speed (m/hour) for elk (*Cervus canadensis*) in southwestern Virginia from 2012 to 2014. EDF is effective degrees of freedom and Ref.df is reference degrees of freedom.

**Parametric Coefficients**				
	Estimate	Error	t value	Pr (>|t|)
(Intercept)	416.390	9.740	42.749	<0.001
year2013	−65.480	11.870	−5.515	0.000
year2014	−57.050	10.270	−5.558	0.000
**Approximate significance of smooth terms**				
	EDF	Ref.df	F	*p*-value
s(julian_date):ageAdult	8.280	8.857	28.191	<0.001
s(julian_date):ageCalf	6.767	7.873	16.049	<0.001
s(julian_date):ageYearling	3.387	4.228	2.405	0.045

**Table A9:** Output from model selection examining weekly net-squared displacement (NSD) from soft-release site (SRS) starting on the date of release for reintroduced elk (*Cervus canadensis*) in southwestern Virginia from 2012 to 2014. We used the Akaike information criterion with a correction for small sample sizes (AICc) to determine which combination of variables best influenced elk movement. The table displays the following information for each model: model formulations, degrees of freedom (DF), AICc values, delta AICc, and the log likelihood (LL). WSR is ‘weeks since release’ and YOR is ‘year of release’.

Model Formulation	DF	AICc	ΔAICc	LL
NSD from SRS ~ s(Julian date, by = year of release) + age class	17.02	−13,313.17	0.00	6673.76
NSD from SRS ~ s(WSR, by = year of release) + age class	19.77	−13,308.92	4.25	6674.45
NSD from SRS ~ s(WSR, by = age class) + YOR	7.67	−13,233.37	79.80	6624.39
NSD from SRS ~ s(Julian date, by = age class) + YOR	7.57	−13,225.62	87.54	6620.41
NSD from SRS ~ s(Julian date, by = YOR)	12.62	−13,207.22	105.94	6616.32
NSD from SRS ~ s(WSR, by = age class)	6.98	−13,206.39	106.78	6610.20
NSD from SRS ~ s(WSR, by = YOR)	9.98	−13,205.86	107.30	6612.96
NSD from SRS ~ s(Julian date, by = age class)	6.67	−13,193.51	119.65	6603.45
NSD from SRS ~ 1	2.00	−13,190.88	122.28	6597.45

**Table A10:** Top model output with approximate significance of smoothing terms for net-squared displacement for elk (*Cervus canadensis*) from the soft release site in southwestern Virginia from 2012 to 2014. The table includes both the parametric model and the smoothed spline model. EDF is effective degrees of freedom and Ref.df is reference degrees of freedom. Std. Error is standard error. WSR is ‘weeks since release’.

**Parametric Coefficients**				
	Estimate	Std. Error	t value	Pr (>|t|)
(Intercept)	6.48 × 10^−4^	6.46 × 10^−4^	1.00	0.317
year2013	−5.10 × 10^−4^	7.78 × 10^−4^	−0.66	0.512
year2014	2.02 × 10^−3^	6.81 × 10^−4^	2.97	0.003
**Approximate significance of smooth terms**				
	EDF	Ref.df	F	*p*-value
s(WSR):ageAdult	1.663	2.070	6.934	0.001
s(WSR):ageCalf	1.000	1.000	0.573	0.449
s(WSR):ageYearling	1.000	1.000	0.003	0.954

**Table A11:** Output from model selection examining weekly mean distance from the soft release site starting on the date of release for reintroduced elk (*Cervus canadensis*) in southwestern Virginia from 2012 to 2014. We used the Akaike information criterion with a correction for small sample sizes (AICc) to determine which combination of variables best influenced elk movement. The table displays the following information for each model: model formulations, degrees of freedom (DF), AICc values, delta AICc, and the log likelihood (LL). WSR is ‘weeks since release’.

Model Formulation	DF	AICc	Delta.AICc	LL
mean.dist ~ s(WSR, by = age) + year	7.00	37,474.95	0.00	−18,730.45
mean.dist ~ s(julian_date, by = year) + year	8.28	37,476.07	1.12	−18,729.72
mean.dist ~ s(WSR, by = year) + year	8.04	37,476.39	1.44	−18,730.11
mean.dist ~ s(julian_date, by = age) + year	8.72	37,481.76	6.81	−18,732.12
mean.dist ~ 1	2.00	37,573.02	98.07	−18,784.51

**Table A12:** Top model output with approximate significance of smoothing terms for mean distance from the soft release site for elk (*Cervus canadensis*) in southwestern Virginia from 2012 to 2014. EDF is effective degrees of freedom and Ref.df is reference degrees of freedom. Std. Error is standard error. WSR is ‘weeks since release’.

**Parametric Coefficients**				
	Estimate	Std. Error	t value	Pr (>|t|)
(Intercept)	1514.300	278.900	5.429	<0.001
year2013	−605.500	335.700	−1.804	0.071
year2014	1360.200	293.900	4.629	<0.001
**Approximate significance of smooth terms**				
	EDF	Ref.df	F	*p*-value
s(WSR):ageAdult	1.000	1.000	0.367	0.544
s(WSR):ageCalf	1.000	1.000	7.912	0.005
s(WSR):ageYearling	1.000	1.000	0.185	0.667
